# Factors associated with the healing of complex surgical wounds in the breast and abdomen: retrospective cohort study

**DOI:** 10.1590/1518-8345.1398.2811

**Published:** 2016-10-10

**Authors:** Eline Lima Borges, José Ferreira Pires, Mery Natali Silva Abreu, Vera Lúcia de Araújo Lima, Patrícia Aparecida Barbosa Silva, Sônia Maria Soares

**Affiliations:** 1 PhD, Adjunct Professor, Escola de Enfermagem, Universidade Federal de Minas Gerais, Belo Horizonte, MG, Brazil.; 2 RN, Hospital das Clínicas, Universidade Federal de Minas Gerais, Belo Horizonte, MG, Brazil.; 3 Specialist in Nursing Care, RN, Hospital das Clínicas, Universidade Federal de Minas Gerais, Belo Horizonte, MG, Brazil.; 4 Doctoral student, Escola de Enfermagem, Universidade Federal de Minas Gerais, Belo Horizonte, MG, Brazil. Scholarship holder from Coordenação de Aperfeiçoamento de Pessoal em Nível Superior (CAPES), Brazil.; 5 PhD, Associate Professor, Escola de Enfermagem, Universidade Federal de Minas Gerais, Belo Horizonte, MG, Brazil.

**Keywords:** Nursing, Wounds and Injuries, Risk Factors

## Abstract

**Objective:**

to estimate the healing rate of complex surgical wounds and its associated factors.

**Method:**

retrospective cohort study from 2003 to 2014 with 160 outpatients of a Brazilian university hospital. Data were obtained through consultation of the medical records. Survival function was estimated using the Kaplan-Meier method and Cox regression model to estimate the likelihood of the occurrence of healing.

**Results:**

the complex surgical wound healing rate was 67.8% (95% CI: 60.8-74.9). Factors associated with a higher likelihood of wound healing were segmentectomy/quadrantectomy surgery, consumption of more than 20 grams/day of alcohol, wound extent of less that 17.3 cm2 and the length of existence of the wound prior to outpatient treatment of less than 15 days, while the use of hydrocolloid covering and Marlex mesh were associated with a lower likelihood of healing.

**Conclusion:**

the wound healing rate was considered high and was associated with the type of surgical intervention, alcohol consumption, type of covering, extent and length of wound existence. Preventive measures can be implemented during the monitoring of the evolution of the complex surgical wound closure, with possibilities of intervention in the modifiable risk factors.

## Introduction

Various international guidelines are available for chronic wounds, such as venous, arterial and pressure ulcers and diabetic foot ulcers, with recommendations for the prevention and treatment of these wounds. For wounds of acute etiology, e.g., surgical wounds, there are few care recommendations, which generates a variety of actions in the practice, without the clarity regarding specific factors that assist or delay the cicatrization process. Associated with this fact, the large number of dressings available, the large number of health professionals involved, and the many opinions regarding effective wound care must be considered^1^.

Surgical wounds (SW) are considered acute, planned and carried out with overlapping edges, which heal by primary intention and have a tendency to regress spontaneously and complete within the expected period. When there is no edge proximity the healing of surgical wounds is by secondary intention. These wounds require more time to heal due to the space between the edges and need greater granulation tissue formation for completion until contraction and epithelialization occur^2^.

It is estimated that 234 million surgeries are performed worldwide each year, with the majority of the SW resulting in healing by first intention^3^. First intention SW can become complex (CSW) when they present complications, such as infection, hematoma and seroma, that cause dehiscence, requiring healing by secondary intention. The incidence of complex surgical wounds described in the literature ranges from 0.5% to 3.0% for adults and 10.0% for older adults, with mortality ranging from 10.0% to 45.0%. The number of new cases of CSW has remained unchanged since the 1950s, despite the scientific advances of the last century^4^ ^-^ ^6^.

In the clinical practice in various Brazilian institutions, doubts still remain about the factors that slow the CSW healing process and which dressings are effective for healing this injury. It is known that the aggravation of the CSW is associated with increased morbidity, mortality and costs to health systems, with hospitalizations and treatments^7^ ^-^ ^8^. Filling this knowledge gap requires development of studies regarding the care of surgical wounds, generating evidence to support more uniform care, prevent undesirable variation in the care and provide better quality of life for the patients.

This study aimed to estimate the complex surgical wound healing rate and identify the factors associated with the healing of these wounds in patients monitored in a Brazilian tertiary hospital.

## Method

This was a retrospective cohort study, involving an outpatient clinic of a large tertiary university hospital of Belo Horizonte, Minas Gerais, Brazil. The sample consisted of patients of both genders, aged over 18 years, undergoing outpatient treatment for CSW in the regions of the breast or abdomen during the period from January 2003 to December 2014. All individuals that met the above criteria were considered potential study participants. Those patients whose respective records contained incomplete information on three or more study variables were excluded from the sample.

Data were obtained by consulting the medical records of the patient, performed by two of the researchers of this study. A semi-structured questionnaire was used for the data collection.

The main variable was the cure (healing) of the complex surgical wound and the secondary variables included sociodemographic data (gender, age, education, family income); behavior (consumption of alcohol, smoking); morbidities (neoplasia, circulatory system disease, diabetes mellitus); neoadjuvant/adjuvant treatment (chemotherapy, radiotherapy); and serum biomarkers (albumin, hemoglobin, fasting glucose). The variables relating to the status of the wound included the number of wounds (1, 2), topography (abdomen, breast), type of intervention surgery, postoperative complications (dehiscence, infection, etc.), beginning of the CSW in the postoperative period, length of existence of the wound prior to outpatient treatment, wound characteristics (length, depth, undermining, exposed point, Marlex screen, necrosis), type of covering (calcium alginate, charcoal with silver, hydrocolloid, foam, hydrofiber).

In the data analysis, a descriptive analysis of the variables used in the study was initially performed by means of frequency distribution tables. The incidences of healing were also estimated for each of the analyzed factors. Survival analysis methodology was used to analyze the time until the occurrence of the healing of the CSW in the studied patients and to compare possible predictors for this event. This technique is used when studying the time until the occurrence of an event of interest (in this case the cure or the healing of the CSW). In this type of analysis, the main characteristic is the presence of censoring, which is the partial observation of the response. That is, for some reason, the monitoring of the patient is interrupted. In this study, censoring occurred due to lack of occurrence of healing by the end of the study, which included death, transfer to another unit and surgery. The survival analysis method makes it possible to incorporate the information contained in the censored data into the statistical analysis^9^.

In the univariate analysis of the data, two survival analysis techniques were used: the first was the Kaplan-Meier method for the construction of survival curves and the second was the Cox proportional hazards model, to estimate the likelihood of healing (hazard ratio), with confidence intervals of 95% (95% CI)^9^.

The Cox regression model was also used in the multivariate analysis. For the input of the predictor variables into the model a *p*-value ≤ 0.20 was used and for permanence of the variable in the final model a 5% significance level was adopted. The adjustment of the final model was estimated from the development of the graph of the logarithm of the survival function versus healing time for each covariable included in the model. Plausible interactions contained in the final model were also tested.

The Statistical Package for the Social Sciences software (SPSS, version 22.0, Chicago, IL, USA) was used for all analyzes of the data.

The project was approved by the Human Research Ethics Committee of the Federal University of Minas Gerais, under authorization No. 01978412.0.0000.5149. As it was a study of medical records, the researchers signed the Terms of Commitment for use of the data.

## Results

A total of 160 medical records of patients with complex surgical wounds in the breast or abdomen region were identified and all met the inclusion criteria. No medical records were excluded due to lack of registration of three or more items. Therefore, 160 individuals and 171 wounds (11 patients had two wounds) were included in this study. The CSW healing rate was 67.8% (95% CI: 60.8-74.9). Of the 52 patients that were discharged without being cured but with area and depth of the CSW reduced, 26 (16.3%) were transferred to the Primary Health Unit, 14 (8.8%) were referred for surgery, either due to cancer relapse or for closure of the CSW by third intention, 11 (6.9%) abandoned the treatment and 1 (0.6%) patient died prior to wound closure.

The mean age of the participants was 48.1 (standard deviation = 15.3) years and 83.8% were female. The sample presented low levels of education and family income, with alcohol consumption above 20 grams/day being observed in 5.0% of the patients and smoking in 22.5%. With regard to the clinical characteristics, the most prevalent morbidities were neoplasia (56.9%) and circulatory diseases (46.9%), radiotherapy neoadjuvant treatment was found in 28.1% of the cases and chemotherapy adjuvant treatment in 24.4% of the cases. There was alterations in serum albumin, hemoglobin and fasting glucose in 62.8%, 43.2% and 34.7% of the participants, respectively (Table 1).

**Table 1:**
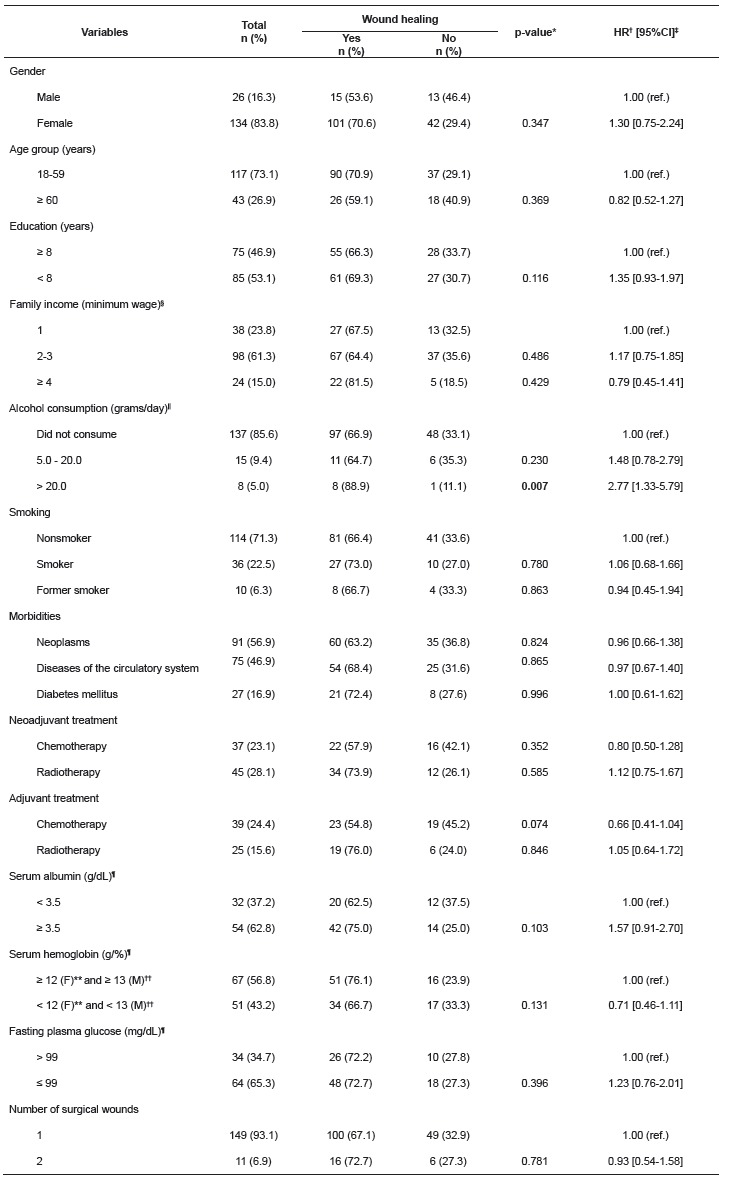
Baseline characteristics according to healing of the complex surgical wound. University Hospital Outpatient Clinic, Belo Horizonte, MG, Brazil, 2003 to 2014

* p-value: differences in the proportions (Cox regression). ^†^HR - hazard ratio; ^‡^Confidence interval. ^§^Minimum wage (Brazil): R$ 240.00 (2003); R$ 260.00 (2004); R$ 300.00 (2005); R$ 350.00 (2006); R$ 380.00 (2007); R$ 415.00 (2008); R$ 465.00 (2009); R$ 510.00 (2010); R$ 540.00 e R$ 545.00 (2011); R$ 622.00 (2012); R$ 678.00 (2013); R$ 724.00 (2014). ^||^Lower alcohol consumption equal to 5.0 grams/day. ^¶^Variations in the total n are due to missing data. ^**^Female. ^††^Male.

Regarding the characteristics of the wounds (Table 2), the most frequent type of surgery was mastectomy (31.3%) and in 93.1% of the cases there was only one wound. In 50.9% of the cases the wound was located on the abdomen and dehiscence was observed in 96.5% of the post-surgical complications. In 52.0% of the cases the CSW came after the 7^th^ day post-surgery and 50.3% of the cases presented 15 days of existence prior to outpatient treatment. The median extension of the wound area was 17.3 cm^2^ (interquartile range 5.7-41.0 cm^2)^ and depth of 1.7 cm (interquartile range from 0.2-3.4 cm). Undermining of the wound occurred in 31.0% of the cases and the point exposed in 23.4%. Marlex mesh was used in 7.6% of patients and necrosis in the wound occurred in 92.4%. The type of cover most used was calcium alginate (80.1%), followed by charcoal with silver (37.4%) and hydrocolloid (33.3%).

**Table 2:**
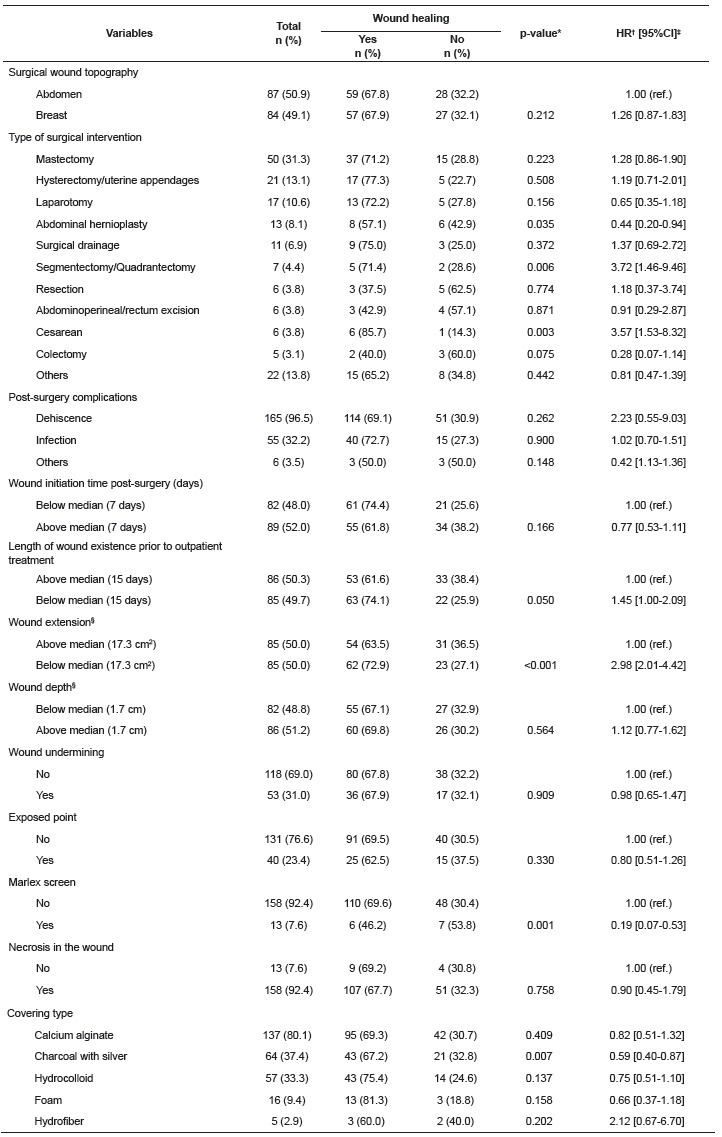
Characteristics of complex surgical wounds according to the outcome variable "cure". University Hospital Outpatient Clinic, Belo Horizonte, MG, Brazil, 2003 to 2014

*p-value: differences in the proportions (Cox regression). †HR - hazard ratio; ‡Confidence interval. §Variations in total n are due to missing data.

**Figure 1:**
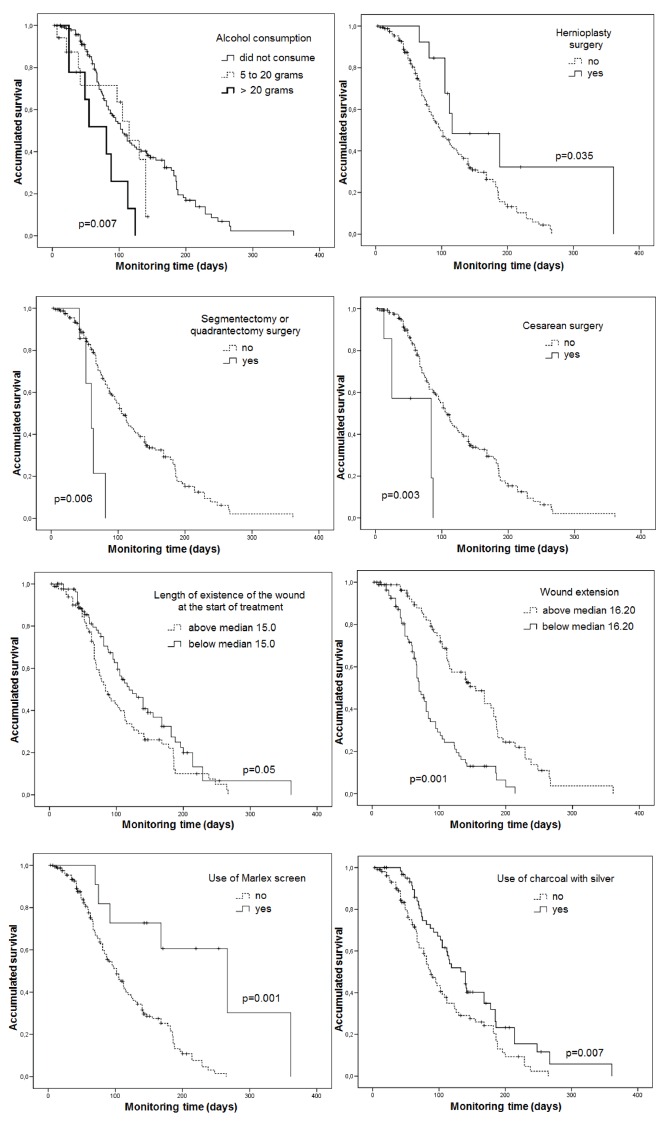
Kaplan-Meier Curves according to basal and clinical characteristics. University Hospital Outpatient Clinic, Belo Horizonte, MG, Brazil, 2003 to 2014

In the univariate analysis (Tables 1 and 2), the following factors were significantly associated with an increased likelihood of curing the CSW in less time (p<0.05): segmentectomy surgery/breast quadrantectomy (hazard ratio [HR] = 3.72; 95% CI = 1.46-9.46), cesarean surgery (HR = 3.57; 95% CI = 1.53-8.32), wound extension less than 17.3 cm^2^ (HR = 2.98; 95% CI = 2.01-4.42), consumption of greater than 20 grams/day of alcohol (HR = 2.77; 95% CI = 1.33-5.79), length of existence of wound prior to outpatient treatment of less than 15 days (HR = 1.45; 95% CI = 1.00-2.09). Other factors were also associated with a lower likelihood of curing the CSW, i.e., longer time for healing: abdominal hernioplasty surgery (HR = 0.44; 95% CI = 0.20-0.94); use of Marlex mesh (HR = 0.19; 95% CI = 0.07-0.53), use of charcoal with silver covering (HR = 0.59; 95% CI = 0.40-0.87 ) in the treatment of the CSW. These results are illustrated by the survival graphs using the Kaplan-Meier method (Figure 1).

According to the results of the multivariate analysis (Table 3), the variables that remained associated with an increased risk of wound healing were segmentectomy/quadrantectomy surgery (HR = 3.38; 95% CI = 1.31-8.69), consumption of more than 20 grams/day of alcohol (HR = 2.71, 95% CI = 1.28-5.74), wound extension of less than 17.3 cm^2^ (HR = 2.47, 95 % = 1.65-3.69) and length of existence of wound prior to outpatient treatment of less than 15 days (HR = 1.54; 95% CI = 1.05-2.25). However, the use of hydrocolloid covering (HR = 0.63; 95% CI = 0.42-0.95) and Marlex mesh (HR = 0.22; 95% CI = 0.08- 0.62) were associated with a lower likelihood of curing the CSW.

**Table 3:** Final adjusted COX proportional hazards model for time until occurrence of the outcome "complex surgical wound healing". University Hospital Outpatient Clinic, Belo Horizonte, MG, Brazil, 2003 to 2014

Variables		HRajusted*	95% CI^†^	p-value^‡^
Segmentectomy/Quadrantectomy				
	No	1.00 (ref.)		
	Yes	3.38	1.31-8.69	0.012
Alcohol consumption (grams/day)^§^				
	Did not consume	1.00 (ref.)		
	5.0 - 20.0	1.67	0.87-3.21	0.120
	> 20.0	2.71	1.28-5.74	0.009
Wound extension				
	Above median (17.3 cm^2)^	1.00 (ref.)		
	Below median (17.3 cm^2)^	2.47	1.65-3.69	<0.001
Length of wound existence prior to outpatient treatment				
	Above median (15 days)	1.00 (ref.)		
	Below median (15 days)	1.54	1.05-2.25	0.028
Hydrocolloid				
	No	1.00 (ref.)		
	Yes	0.63	0.42-0.95	0.028
Marlex screen				
	No	1.00 (ref.)		
	Yes	0.22	0.08-0.62	0.004

* HR - hazard ratio. ^†^Confidence interval ^‡^differences in the proportions (Cox regression). ^§^Lower alcohol consumption equal to 5.0 grams/day.

The adjustment of final model was satisfactory, according to the graphical interpretation of the logarithm of the survival function versus time, indicating that the proportional odds assumption was not violated. There was no interaction verified between the covariates included in the final model.

## Discussion

The CSW has a great impact on the quality of life of patients due to the pain, unpleasant odor and exudate from the lesions, which can be associated with sleep problems, restriction of activities and leisure, reduced productivity at work and social isolation, in addition to economic burdens due to the treatment of the injury^10^.

This is the first retrospective cohort study published nationally or internationally that estimated the complex wound healing rate and associated factors with outpatients. Due to lack of specific studies on CSW, comparisons of data with other investigations, at times, were extrapolated to chronic wounds.

The healing rate in this study is similar to data from the United States, considering all types of wounds^7^. A retrospective study, based on data from the US Wound Registry, indicated that 20.8% of all types of wounds are represented by unhealed surgical wounds. The mean cost for a healing wound was 3,927 US dollars. The cost of unhealed wounds increases with longer duration of outpatient monitoring, indicating increased complexity of the wound. Some factors that contribute to increasing the cost of wound healing include diabetes mellitus, use of systemic antibiotics, chronic kidney disease, immunosuppressive drugs, smoking and multiple morbidities^7^.

Median hospital costs can reach 14,094 US dollars per patient for slight complications in the SW and 28,356 US dollars for those with major complications^8^. A phase II intervention study of a protocol to reduce the incidence of complications in SW in obese gynecological oncology patients, conducted in the United States, estimated an increase of at least 3,500 US dollars in the surgical procedures due to the treatment of CSW^11^.

The high cost associated with patients with chronic wounds was also reported in Wales, UK. The total cost was approximately 328.8 million pounds, a mean of 1,727 pounds sterling per patient, accounting for 5.5% of the National Health Service (NHS) spending^12^.

Regarding the predictors associated with healing of the CSW, consumption of more than 20.0 grams of alcohol was a protective factor for wound healing, contradicting previous studies. In a systematic review with meta-analysis, the preoperative consumption of alcohol was associated with an increased risk of postoperative morbidity in general, including wound complications (HR = 1.23; 95% CI = 1.09-1.40). In the same study, low and moderate preoperative alcohol consumption did not seem to be associated with the occurrence of complications in the postoperative period^13^. However, an experimental study with rats showed that acute alcohol intoxication did not alter the healing of colonic anastomosis wounds, although it increased the death rate in the postoperative period^14^. From this finding, the authors plan to study the effect of alcohol on wound healing through animal experiments in order to clarify these results.

Among the surgeries, segmentectomy/quadrantectomy was a secondary variable that presented a higher likelihood of wound healing. This may be explained due to the lower complexity of the surgery and the involvement of a smaller area of extension in the surgery. Quadrantectomy is performed after diagnosis of breast cancer, in which the tumor is removed with clear margins (from the skin to the muscle) and the rest of the breast is maintained. In general this operation is possible when there are small tumors less than 3 cm, and a good tumor to breast ratio. At this time, segmentectomy is minor breast surgery, used to confirm the diagnosis (e.g., in the presence of microcalcifications, hemorrhagic papillary flow)^15^. Similarly, the smaller extent of the wound has been associated with an increased rate of wound healing, considering that less formation of granulation tissue and reepithelialization are required. The length of existence of the wound prior to outpatient treatment also acted as a protective factor in the wound healing. One possible explanation is that acute wounds tend to heal faster when compared with chronic wounds^16^. Another factor that may contribute to the result is the more rapid institution of the therapeutic treatment, with the prognosis for wound closure being better as possible complications can be ameliorated.

Conversely, the Marlex screen delayed wound healing. This occurred due to it being a foreign body, triggering a reaction in the tissues, which could vary from an exudative process, a foreign body granuloma reaction, to a hypersensitivity immune response of the patient and formation of adhesions^17^. Another experimental study with rats showed that the use of polypropylene mesh surrounded by fibrous tissue was more effective in the correction of induced abdominal hernias, with a lower degree of macroscopic adhesions when compared to polypropylene mesh^18^.

Despite the extensive literature^19^ ^-^ ^20^ demonstrating the use of hydrocolloid as an effective wound closure covering, in this study it was negatively associated with the healing process. This is because patients who used hydrocolloid, also initially made use of charcoal with silver, to reduce the bacterial load of the lesions with critical colonization or infection, indicating that these were patients with greater wound severity^21^ that required more time for the healing of the wound.

One limitation of this study refers to the collection of data from secondary sources, not always adequately completed or present. This fact contributed to some variables not being analyzed, such as those related to the surgical technique (tension in the surgical wound, type of asepsis and antisepsis, type of thread used to suture) and the body mass index of the patients. Another limitation to be highlighted is the lack of a control group of individuals without surgical wound complication for comparison of the results.

Despite this being a longitudinal study, some questions can be raised: would the prospective cohort study design involve other variables related to the intrinsic and extrinsic factors of the person that could interfere with the healing process? In addition, other types of study could answer the question regarding the consumption of 20 grams/day of alcohol being associated with an increased likelihood of healing CSW.

These questions highlight the need for further studies with different methodological approaches to verify the associations with the likelihood of curing the CSW.

## Conclusion

The CSW healing rate was high, considering the length of monitoring and the various factors that interfere with the healing process. Type of surgical procedure, alcohol consumption, type of covering, wound extent and length of existence of the wound were associated with the outcome. Knowledge of this risk profile for curing the CSW will allow health professionals to adopt preventive measures during the monitoring of the evolution of the wound closure, with possibilities of intervention in the modifiable risk factors.
